# Lignin-Based Coatings: A Sustainable Approach to Produce Antibacterial Textiles

**DOI:** 10.3390/ijms26031217

**Published:** 2025-01-30

**Authors:** Sílvia Ferreira, Vânia Pais, João Bessa, Fernando Cunha, Laura de Araújo Hsia, Estevão Frigini Mai, Giullia Sborchia, Raul Fangueiro

**Affiliations:** 1 Fibrenamics—Institute of Innovation on Fiber-Based Materials and Composites, University of Minho, 4800-058 Guimarães, Portugal; vaniapais@fibrenamics.com (V.P.); joaobessa@fibrenamics.com (J.B.); fernandocunha@fibrenamics.com (F.C.); rfangueiro@fibrenamics.com (R.F.); 2 Suzano S.A., São Paulo 01452-002, Brazil; laurahsia@suzano.com.br (L.d.A.H.); estevao.mai@suzano.com.br (E.F.M.); giullias@suzano.com.br (G.S.); 3 Department of Textile Engineering, Centre for Textile Science and Technology (2C2T), University of Minho, 4800-058 Guimarães, Portugal

**Keywords:** lignin, paper industry, antimicrobial textiles, knife-coating, cotton and polyester fabrics

## Abstract

The growing interest in developing antibacterial textiles using natural functional agents is largely driven by their sustainable and eco-friendly attributes. Lignin, a highly available biopolymer with a polyphenolic structure, has drawn attention due to its potential as a bioactive antibacterial agent. However, its inherent heterogeneity poses challenges, particularly regarding its antibacterial efficacy. In this study, unmodified kraft lignin sourced directly from the paper industry was applied to cotton and polyester fabrics, using a knife-coating technique with varying concentrations (0%, 5%, 10%, 20%, and 30% *w*/*v*), to assess its potential as an antibacterial coating. The lignin-coated fabrics demonstrated hydrophobic properties, with water contact angles reaching up to 110.3° and 112.6°, for polyester and cotton fabrics, respectively, alongside significantly reduced air permeability and water vapor permeability indexes, regardless of lignin concentration. Antibacterial evaluations also revealed that lignin-based coatings, with at least 10% *w*/*v* concentration, allowed cotton fabrics with a bacterial reduction surpassing 96%, according to ASTM E2149-2013, particularly for Gram-positive *S. aureus*, highlighting the potential of lignin as an antibacterial agent. Despite their limited resistance to domestic washing, the lignin-coated fabrics demonstrated exceptional stability under hot-pressing conditions. Therefore, this stability, combined with the hydrophobic and antibacterial properties observed, particularly on coated cotton fabrics, highlights the potential application of lignin-based coatings for the development of antibacterial and water-repellent textiles, with these coatings being particularly suited for single-use applications or scenarios where washing resistance is not a requirement. This approach offers a sustainable and efficient method for producing functional textiles while enabling value-added utilization of lignin, showcasing its potential as an eco-friendly solution in textile functionalization.

## 1. Introduction

The increasing focus on environmental sustainability has driven substantial research and innovation in the textile industry, particularly in the development of functional textiles with minimal environmental impact [1]. Among these, antibacterial textiles have earned significant attention due to their wide range of applications across multiple sectors, including healthcare (scrubs, masks, lab coats), apparel (sportswear, underwear, outerwear, winter wear), households (bedding, covers, pillows, towels), personal protective equipment (PPE), or environmental and public health [2,3,4]. Owing to their inherent properties, these textiles are capable of inhibiting the growth, development, and proliferation of microorganisms, preventing microbial contamination and infection [2]. Currently, conventional strategies for imparting antimicrobial properties to textiles predominantly rely on the incorporation of synthetic agents, such as quaternary ammonium compounds, metallic nanoparticles, triclosan, or N-Halamine [2,3,5]. Despite their high efficacy in providing rapid antimicrobial action, these agents face growing scrutiny due to their environmental persistence, potential toxicity, and dependence on non-renewable resources. As a result, there is an increasing demand for more sustainable alternatives capable of delivering comparable antimicrobial efficacy while minimizing ecological and health-related risks [5].

In the search for greener alternatives, lignin has emerged as a promising alternative to the current antimicrobial agents [6]. As the second most abundant natural polymer on Earth, lignin is a key structural component of plant cell walls, providing mechanical strength and accounting for 10–30% of biomass by weight [7,8]. Despite its widespread availability, most commercial lignin is a byproduct of industrial processes focused on cellulose extraction, such as the pulp and paper industry and bioethanol production. However, only a small fraction (1–2%) of lignin is valorized into high-value products, with the bulk being incinerated for energy recovery [9,10].

However, due to their highly branched and aromatic structure, lignin is rich in functional groups including hydroxyl, methoxyl, carbonyl, carboxyl, and quinone groups. This composition has recently highlighted lignin as a novel bioactive natural polymer with multiple applications—not only as an antimicrobial, but also as an antioxidant, flame retardant, water-repellent, and ultraviolet (UV)-blocking agent [8,11]. Given the natural variability of lignin, significant attention has now been directed to understand the efficacy of these functionalities according to its source and the isolation processes and parameters applied, as well as how they can be manipulated to enhance their effect [9,12,13,14,15]. For instance, Yun et al. [16] demonstrated that low-molecular-weight lignin extracted from bamboo via the kraft process effectively inhibited the growth of both Gram-negative bacteria (*Escherichia coli* and *Salmonella*) and Gram-positive bacteria (*Streptococcus* and *Staphylococcus aureus*) by disrupting the bacterial cell wall. Similarly, Gordobil et al. [17] reported that lignin from spruce and eucalyptus, obtained through organosolv extraction, exhibited not only antibacterial activity against *Aspergillus niger* and *Salmonella enterica,* but also an enhanced sun protection factor (SPF).

Considering these promising bioactive properties, lignin and its derivatives have gained increasing interest as natural antimicrobial agents for textile applications. To achieve antimicrobial properties in textiles, various approaches have been adopted, ranging from the incorporation of lignin during the production of textile fibers [18] to the functionalization of textile structures using different types of techniques [6,19]. Regarding this last topic, several authors have been exploring the extraction of lignin from different sources, such as black liquor [20], agricultural waste [21,22], and food residues [23], followed by its dissolution in organic solvents, like dimethyl sulfoxide (DMSO), and subsequent application to textile substrates. These methods have demonstrated significant antibacterial efficacy against both Gram-positive and Gram-negative bacteria. Additionally, through the modification of lignin, multifunctional cotton fabrics were also prepared by covalent grafting of phosphorylated lignin, which not only conferred antibacterial properties, but also enhanced fire resistance, with excellent durability [24]. In an attempt to increase the effectiveness of the functionalities and using more sophisticated methods, recent approaches have also focused on exploring the application of lignin as nanoparticles [25] or in combination with other bioactive agents, such as chitosan, applied through layer-by-layer deposition, or metallic nanoparticles [26,27,28,29]. These approaches have led to substantial improvements not only in antibacterial performance, but also in the antioxidant and UV-protection capabilities of the treated textiles.

Building on this, the present study aims to investigate the potential of unmodified lignin, sourced from the paper industry, as an antibacterial agent for textiles, in particular, cotton and polyester fabrics. These will be achieved through knife coating—an efficient, cost-effective, and widely used technique in the textile industry. By varying the concentration of the lignin in the coating’s formulation, the goal is to assess which concentration allows the development of efficient antibacterial textile fabrics against both Gram-positive and Gram-negative bacteria, while also evaluating key textile properties, such as air and water vapor permeability and wettability. Additionally, this study examines the durability of the coatings through multiple domestic washing and hot-pressing cycles to evaluate the durability of the functional properties. Therefore, this research aims to contribute to the growing knowledge of sustainable materials in the textile industry, highlighting lignin as a renewable resource capable of producing high-performance antibacterial textiles with a reduced environmental footprint.

## 2. Results and Discussion

### 2.1. Characterization of Coated Fabrics

**Figure 1:**
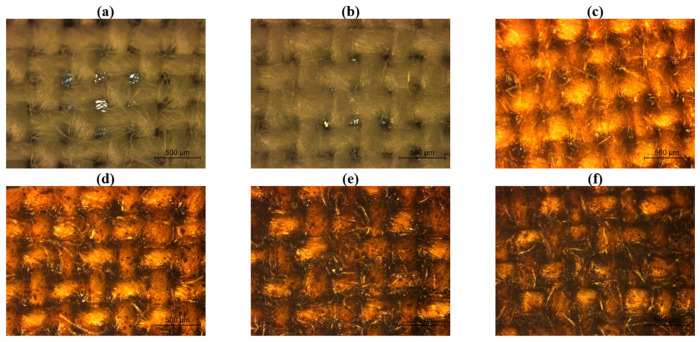
Cotton fabrics observed under a microscope at a total magnification of 50: (**a**) without coating, and coated with (**b**) 0%, (**c**) 5%, (**d**) 10%, (**e**) 20%, and (**f**) 30% lignin.

**Figure 2:**
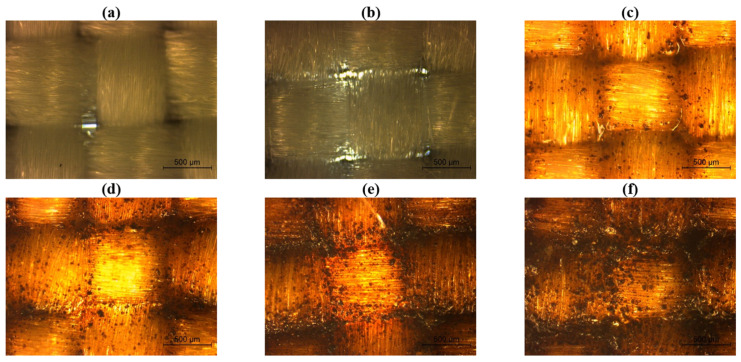
Polyester fabrics observed under a microscope at a total magnification of 50: (**a**) without coating, and coated with (**b**) 0%, (**c**) 5%, (**d**) 10%, (**e**) 20%, and (**f**) 30% lignin.

To evaluate the effect of lignin coatings at different concentrations on the surface morphology of cotton and polyester fabrics, optical microscopy analysis was conducted. As demonstrated in Figure 1 and Figure 2, a clear progressive modification in the fabric’s surface was observed as the lignin concentration increased. Regarding the cotton samples (Figure 1), in comparison to the uncoated fabric and the sample coated only with the polymeric base (0% lignin), the introduction of lignin at a 5% concentration led to a noticeable change in fabric coloration. As the lignin concentration rises to 10% and 20%, lignin particles have become increasingly apparent on the fabric surface, predominantly accumulating at the intersections of the warp and weft yarns, which reduced the air gaps initially observed in the fabric structure. At the highest concentration (30%), the fabric surface was extensively coated with lignin, significantly affecting the visual appearance of the surface fabric.

In the case of polyester samples, a similar behavior was also observed. As illustrated in Figure 2, the incorporation of 5% lignin resulted in a perceptible alteration in the surface color of the fabric, accompanied by the detection of discrete lignin particles, mainly in the intersections of the warp and weft yarns. With the progressive increase in lignin concentration, the presence of these particles became increasingly prominent, leading to a gradual masking of the underlying polyester fabric, this effect being particularly pronounced in the fabric coated with 30% lignin. Nevertheless, despite the higher lignin content, lignin particles were distributed across the entire fabric’s surface.

**Figure 3:**
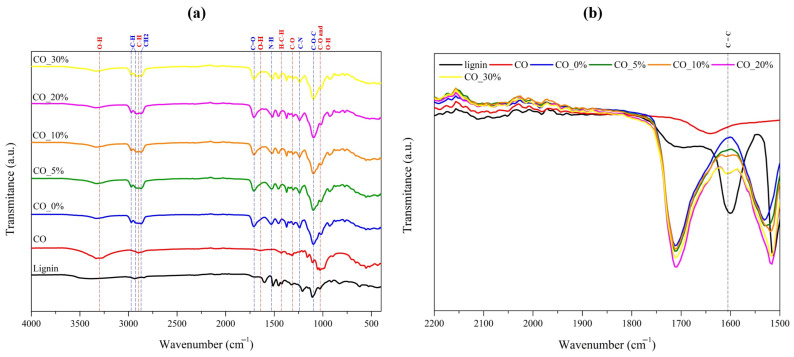
FTIR spectrum of uncoated and coated cotton fabrics ranging from (**a**) 4000 to 400 cm⁻^1^ and (**b**) 2200 to 1500 cm^−1^. (--- represents characteristic peaks of chemical bonds present in cotton fabric, --- represents characteristic peaks of chemical bonds present in polyurethane polymer base, and --- indicates the C=C bond peak from lignin).

Along with the morphological assessment, the chemical composition of the samples was also examined through FTIR. Concerning the cotton samples, and as shown in Figure 3a, the uncoated cotton (CO) FTIR spectrum exhibited distinct broad peaks at 3000–3500 cm^−1^ (O–H stretching vibration), 2901 cm^−1^ (C–H stretching vibration), 1635 cm^−1^ (O–H bending vibration), 1430 cm^−1^, (H-C-H vibration), 1317 cm^−1^ (C-O vibration), and 1036 cm^−1^ (combination of C–O and O-H vibration), which are characteristic of cellulose, the main component of cotton [30,31]. After the application of polymeric coating without lignin (CO_0%), new peaks emerged related to chemical bonds present in the polyurethane polymeric base used. Therefore, the FTIR spectrum of CO_0% presented new peaks, at 2963 cm^−1^, 2930 cm^−1^, 2867 cm^−1^, 1712 cm^−1^, 1530 cm^−1^, 1235 cm^−1^, and 1095 cm^−1^, which are related to the vibration of the chemical bonds C–H of the aliphatic chain, –C–H, –CH_2_–, C=O, N-H, C-N, and C-O-C, respectively [19,32]. The incorporation of lignin was only detected after a more detailed analysis of the FTIR spectrums (Figure 3b), which revealed the emergence of a new peak on the lignin-coated fabrics (CO_5%, CO_10%, CO_20%, and CO_30%), around 1600 cm⁻^1^. This peak corresponds to the stretching vibration of C=C bonds, a chemical bond only present in the chemical structure of lignin [33]. Additionally, upon comparing the FTIR spectrums of lignin-coated cotton fabrics, an increase in this peak intensity was also observed, mainly for high lignin concentrations. This indicates not only the successful incorporation of lignin into the coatings, but also the increasing amount of lignin as a function of its concentration in the coating paste formulation.

**Figure 4:**
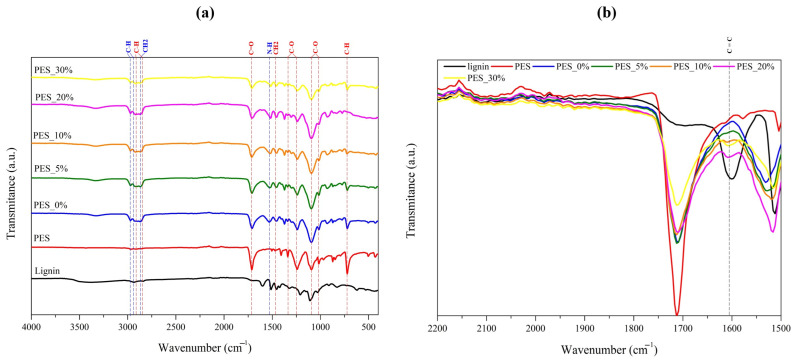
FTIR spectrum of uncoated and coated polyester fabrics ranging from (**a**) 4000 to 400 cm⁻^1^ and (**b**) 2200 to 1500 cm^−1^. (--- represents characteristic peaks of chemical bonds present in polyester fabric, --- represents characteristic peaks of chemical bonds present in polyurethane polymer base, and --- indicates the C=C bond peak from lignin).

As can be seen in Figure 4a, in the case of polyester fabric samples, slightly different peaks were detected. Therefore, for the uncoated polyester fabric (PES), peaks were identified at 2924–2848 cm^−1^ (symmetric and asymmetric vibrations of C–H from alkyl chains), 1710 cm^−1^ (stretching vibration of C=O), 1460 cm^−1^ (bending of CH_2_), 1338 and 1239 cm^−1^ (stretching vibration of O–C, from acid), 1093 and 1012 cm^−1^ (stretching vibration of O–C, from ester), and at 700 cm^−1^ (bending and ring puckering vibrations of the C–H on the benzene rings), all related with the chemical structure of polyester fabric [34]. After applying the coating without lignin (PES_0%), and contrary to what was observed in the cotton samples, only a few peaks referring to the chemical bonds of the polymeric polyurethane base were detected, namely, at 2963 cm^−1^, 2930 cm^−1^, 2867 cm^−1^, and 1530 cm^−1^, which are related with the vibration of the chemical bonds C–H of the aliphatic chain, C–H, CH_2_–, and -N-H, respectively [19,32]. This is related to the fact that there are common chemical bonds or bonds that vibrate at the same wavenumber in polyester and polyurethane. As observed in the cotton samples, the presence of lignin in the lignin-coated polyester fabrics (PES_5%, PES_10%, PES_20%, and PES_30%) was also identified upon a more detailed analysis of FTIR spectrums, as presented in Figure 4b. This analysis revealed a new peak near 1600 cm^−1^, corresponding to the C=C stretching vibration, a chemical bond that is only present in the lignin chemical structure. Thus, the identification of this peak not only confirms the successful incorporation of lignin into the coatings applied to polyester fabrics, but also provides evidence of a concentration-dependent increase in lignin content, as the intensity of the peak progressively rises with higher lignin concentrations. These findings, corroborated by previous microscopy observations, validate the successful application of coatings with different lignin concentrations on both cotton and polyester fabrics.

**Figure 5:**
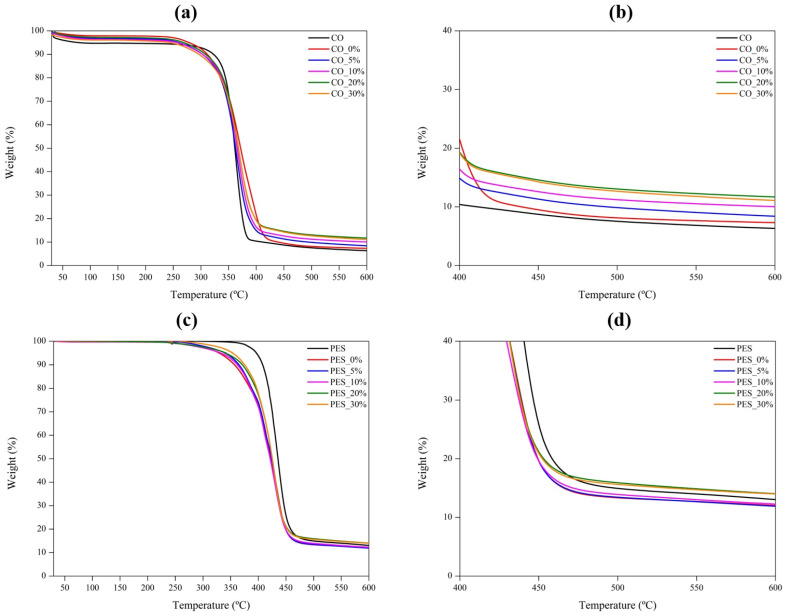
TGA curves of uncoated and coated fabrics: (**a**,**b**) represent cotton fabric samples, and (**c**,**d**) represent polyester fabric samples. The temperature ranges are as follows: (**a**,**c**) from 25 °C to 600 °C, and (**b**,**d**) from 400 °C to 600 °C.

As one of the objectives of this study was to evaluate the durability of the coatings, particularly when subjected to hot-pressing conditions, the thermal stability of both cotton and polyester fabric samples was analyzed by thermogravimetric analysis. According to the TGA curves of cotton samples presented in Figure 5a, all samples exhibited three distinct stages of weight loss. The initial one, which does not exceed 5%, occurred up to 100 °C and is primarily attributed to the gradual evaporation of the adsorbed water. Between 200 °C and 400 °C, the most significant weight loss was observed, corresponding to the thermal degradation of the main components present in the samples, with the degradation rate slowing down progressively to 600 °C [20]. For the uncoated cotton fabric (CO), thermal degradation began around 300 °C, presenting a total weight loss of 97.7% at 600 °C. However, when the coating is applied, the onset of thermal degradation shifts to a lower temperature, approximately 250 °C, which can be attributed to the beginning of polyurethane polymer base degradation, which starts around this temperature [35]. The incorporation of lignin into the coating does not significantly alter the onset temperature, as its thermal degradation also started at approximately 250 °C [33]. Nonetheless, differences were observed in total weight loss up to 600 °C, as can be seen in Figure 5b. As the lignin concentration in the coating increased, a proportional decrease in weight loss was observed at 600 °C, with values of 91.6%, 90%, 88.3%, and 88.9% for the samples CO_5%, CO_10%, CO_20%, and CO_30%, respectively. This reduced weight loss can be primarily attributed to the slower thermal degradation rate of lignin as well as its highly aromatic structure, which promotes the formation of a substantial amount of char residue. This char formation acts as a thermal barrier, reducing the overall weight loss of lignin-coated samples at 600 °C, when compared to uncoated cotton (CO) or fabrics coated exclusively with the polymeric base (CO_0%) [6,33].

For polyester samples, the thermal behavior was slightly different. As observed in the TGA curves presented in Figure 5c, the uncoated polyester fabric showed thermal stability up to approximately 300 °C, with no weight loss detected below this temperature. This can be attributed to polyester’s low moisture adsorption capacity, which prevents early-stage weight loss associated with water evaporation. The most pronounced thermal degradation occurred between 350 °C and 500 °C, corresponding to the breakdown of the polymer chains, particularly the ester bonds [36]. Beyond 500 °C, the degradation rate progressively slowed, and by 600 °C, the total weight loss of the uncoated polyester fabric reached 87.1%. After the application of coatings, the thermal degradation profile was altered. The onset of thermal degradation shifted to a lower temperature of approximately 250 °C, consistent with the initial decomposition of the polyurethane polymer base [35]. The effect of lignin in the applied coatings was more pronounced only for higher concentrations, namely, 20% and 30%, where the thermal degradation was slower, leading to a lower weight loss at 600 °C, with values around 86%, as can be seen by Figure 5d. As previously mentioned, this behavior can be related to the slower thermal degradation rate of lignin, as well as its highly aromatic structure, which promotes the formation of a substantial amount of char residue, improving the thermal stability of the coated fabric and reducing the overall weight loss of lignin coated samples [6,33].

### 2.2. Permeability and Contact Angle Evaluation of Coated Fabrics

Nowadays, antibacterial textiles are used in a variety of applications ranging from households to commercials including air filters, food packaging, health care, apparel, hygiene, medical, sportswear, storage, ventilation, and water purification systems [2]. Therefore, to assess the potential application of the lignin-coated cotton and polyester fabrics, their comfort properties were evaluated, by analyzing air and water vapor permeability, as well as wettability through water contact angle measurements.

Air permeability is one of the fundamental fabric properties that provides critical insights into comfort, particularly for wearable applications. This property quantifies the air flow through the fabric under specified pressure differentials between the front and back surfaces [37]. According to the results presented in Table 1, uncoated cotton fabric (CO) exhibited the greatest permeability to air (679.8 L/m^2^/s) among the tested samples. The high air permeability suggests that air can easily flow through the pores of the cotton fabric. In contrast, after the application of coating on both sides of the fabric, the air permeability drastically decreased, yielding values significantly lower (*p* < 0.0001), between 1.3 and 2.7 L/m^2^/s, as presented in Table 1. This effect is already reported in the literature for PU-coated textiles [38]. Considering that air permeability is largely influenced by textile openness as well as its structure and composition, the application of coatings, with or without the incorporation of lignin, completely blocked the air gaps present in the cotton fabric, reducing significantly the air flow through the fabric [38,39]. No statistically significant differences were observed between coated cotton samples (*p* > 0.05). In the case of the polyester samples (Table 2), it was observed that the uncoated fabric exhibited an air permeability of 57 L/m^2^/s, which is significantly lower than the value obtained for cotton fabric. This can be attributed to the higher linear density of the polyester fabric used, which resulted in a denser structure with fewer air gaps, restricting the air flow. The further application of a coating on both sides of the fabric obstructs its air passages, leading to a significant reduction in air permeability (*p* < 0.0001), with values ranging from 2.3 to 7.8 L/m^2^/s, as presented in Table 2. This decrease in permeability underscores the effect of both the inherent properties of polyester fabric and the applied coating, which cumulatively reduces the fabric’s air permeability [38,39]. In the coated polyester samples, the addition of lignin at different concentrations resulted in statistically significant differences (*p* < 0.001) compared to the coated sample without lignin (PES_0%). Incorporating 5% to 20% lignin into the coating further reduced the air permeability of the fabric, likely due to a more effective blockage of air gaps. At a lignin concentration of 30%, the air permeability increased to 7.8 L/m^2^/s. This increase may be attributed to the higher lignin content, potentially causing particle agglomeration or the formation of microfissures in the coating, which could partially restore some air flow through the fabric. However, not to the extent of considering the fabrics permeable.

The breathability of a fabric, defined as its capacity to allow moisture vapor transmission, was assessed through the measurement of its water vapor permeability (WVP) index. As shown in Table 1, the uncoated cotton (CO) exhibited the highest WVP index, 100%, indicating excellent breathability and efficient moisture vapor passage through its porous structure. Following the application of coatings, the WVP index of coated cotton fabrics decreased significantly (*p* < 0.0001), with values ranging between 34% and 44%. Additionally, in the coated cotton samples, the addition of coatings with 10% and 30% lignin significantly lowered even more the WVP index, when compared with CO_0% (*p* < 0.05). Given that a minimum WVP index of 50% is typically required for a fabric to be classified as breathable [39], all the coated cotton fabrics in this study do not meet the criteria for breathable textiles. However, despite the extremely low air permeability values observed in the coated structures, which effectively block air flow, these fabrics still exhibit a measurable water vapor permeability, which demonstrates that the coated fabrics retain the capacity for limited water vapor transmission, although not to an extent to classify the structures as breathable. On the other hand, the uncoated polyester fabric (Table 2) exhibited a WVP index of 84%, which was lower than that observed for uncoated cotton fabric. This can be attributed to the more compact structure of polyester fabric, which reduced the passage of water vapor. Upon the application of coatings, a significant reduction in the WVP index was observed (*p* < 0.0001), with values ranging from 9.2% to 12.0%. Additionally, no statistically significant differences were observed between coated polyester samples (*p* > 0.05). Therefore, the coated polyester fabrics cannot be classified as breathable, as their WVP indexes are well below the 50% threshold typically associated with breathable fabrics. In comparison with the coated cotton samples, the coated polyester fabrics showed substantially lower WVP values, indicating their limited ability to allow water vapor transmission, which adversely affects their comfort properties. This reduction in breathability highlights the potential limitations of coated polyester in applications where moisture management and comfort are critical.

**Figure 6:**
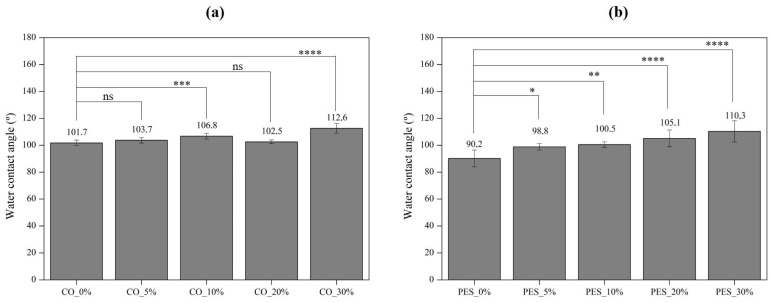
Water contact angle of coated (**a**) cotton and (**b**) polyester fabrics, (*n* = 10, ±SD), ns (non-significant), *p* > 0.05, * *p* < 0.05, ** *p* < 0.01, *** *p* < 0.001, **** *p* < 0.0001 (one-way ANOVA, Šídák’s test).

In addition to permeability, the wettability of the coated fabrics was also analyzed by measuring the static water contact angle, which reflects the interaction between a solid surface and a liquid, in this case, water. Therefore, when the contact angle is higher than 90°, the solid does not have affinity with the liquid, and the material is classified as hydrophobic. Otherwise, if the angle is lower than 90°, the affinity with the liquid is higher and the material is classified as hydrophilic [40]. Based on this, the contact angle values for cotton and polyester samples are represented in Figure 6a and Figure 6b, respectively. The water contact angle of both the uncoated cotton (CO) and polyester (PES) fabric is not presented since the water drop was instantly absorbed, making it impossible to measure the contact angle. This rapid absorption not only confirms the hydrophilic nature of cotton, as consistently reported in the literature [37], but also the hydrophilic behavior of the polyester fabric used in this study.

Therefore, based on the contact angles obtained for coated cotton fabrics (Figure 6a), it was observed that all samples exhibited hydrophobic behavior following the application of the coating, with static contact angles between 101.7° and 112.6°. This significant change can be primarily attributed to the hydrophobic nature of the polyurethane polymeric base used as the coating base, which lowers the overall surface energy of the cotton fabric [37]. Additionally, the incorporation of lignin, particularly at a 10% (CO_10%) and 30% concentration (CO_30%), resulted in a significant increase in the contact angle, when compared to the coated fabric without lignin (CO_0%), reaching contact angle values of 106.8° and 112.6°, respectively. This result suggests that, in addition to the polymeric base, the inclusion of lignin further enhanced the hydrophobic properties of the coated fabric, inhibiting the spread of water droplets on the surface. The increase in contact angle values observed with the incorporation of lignin has been reported in previous studies and attributed to lignin’s complex chemical structure, which includes hydrophobic chemical groups as well as changes in surface roughness and a reduction in surface energy [38,41,42].

Similarly, the wettability of the polyester fabrics underwent notable changes after the coating application. As can be seen in Figure 6b, all coated polyester fabrics presented a water contact angle equal to or greater than 90°, therefore being classified as hydrophobic. As with the cotton samples, this change in wettability can be explained by the hydrophobic nature of the polyurethane polymeric base used, combined with the lignin incorporation, which increased even more the water contact angles [37,38]. The addition of lignin to the coating led to a gradual significant increase in the contact angle as a function of the concentration applied, reaching a maximum water contact angle of 110° for PES_30%. These findings demonstrate that the lignin-based coatings impart strong hydrophobic performance to the cotton and polyester fabrics.

Based on wettability and permeability results, the application of lignin-based coatings has resulted in the development of hydrophobic cotton and polyester fabrics that are almost impermeable to air and exhibit reduced breathability, especially coated polyester fabrics. Given these properties, such materials show some potential for applications in water-repellent or windproof textiles, especially where breathability is not a critical requirement [39,40].

### 2.3. Antibacterial Properties of Coated Fabrics

One of the main goals of this study was to investigate the feasibility of producing antibacterial textiles through the application of unmodified lignin, sourced directly from the paper industry, as a coating in cotton and polyester fabrics. Therefore, to evaluate the antibacterial efficacy, both Gram-positive (*Staphylococcus aureus*) and Gram-negative (*Klebsiella pneumoniae*) bacteria were used, applying dynamic contact conditions, according to ASTM E2149-2013. The antibacterial activity was first evaluated for all the cotton samples and then, based on its performance, only specific polyester samples were analyzed. The results are presented in Table 3 and Table 4.

Regarding the cotton samples, for the uncoated cotton (CO) and the cotton coated without lignin (CO_0%), no antibacterial activity was observed against both bacterial strains. In these samples, the number of bacteria increased 10^6^ and 10^7^ CFU/mL, respectively, when compared to the inoculum at “zero” time, indicating significant microorganism proliferation and, therefore, no antibacterial effect. However, with the incorporation of lignin into the coating, the cotton fabrics began to exhibit an antibacterial effect, particularly against *S. aureus*. Concentrations of lignin equal to or greater than 10% in the coating demonstrated strong antibacterial activity, with bacterial reduction (*R*) values reaching 97.3%. These results indicate that the number of bacteria in lignin-coated samples was not only lower than the number of bacteria present in the inoculum (control) but was also equal to or even lower than the number of bacteria at the beginning of the test, suggesting the coatings’ bactericidal properties and their ability to prevent bacterial proliferation [6]. According to the available studies, this antibacterial effect is related to lignin’s polyphenolic structure, which has the capacity to interact with bacteria’s cellular membrane, changing its metabolism and consequently preventing its proliferation [6,9,12]. In contrast, the same antibacterial effect was not observed against *K. pneumoniae*, regardless of the amount of lignin applied to the coating. As shown in Table 3, the bacterial counts on all lignin-coated fabrics remained in the same order of magnitude or even higher than the inoculum after 24 h of contact, leading to low or even zero reduction values. Additionally, compared to the initial bacterial count at “zero” time, an exponential growth was observed, clearly indicating that the lignin-coated fabrics did not inhibit the proliferation of this Gram-negative bacteria.

Considering these findings, the antibacterial performance of polyester samples was subsequently assessed, focusing especially on the uncoated polyester fabric (PES), as well as the coated fabrics PES_0% and PES_10%.

As seen in Table 4, for both uncoated polyester (PES) and the polyester coated without lignin (PES_0%), no antibacterial activity was observed against *S. aureus* and *K. pneumoniae.* The bacterial count increased by more than 10^7^ CFU/mL in both samples, when compared to the inoculum at “zero” time, indicating significant microorganism proliferation, and suggesting that neither the uncoated polyester fabric nor the coated polyester without lignin had any effective antibacterial properties. After the application of 10% lignin into the coating, the coated polyester fabric presented a higher bacterial reduction (84.3%), against *S. aureus*. This result indicates that the bacterial count was 5.8 × 10^6^ CFU/mL lower than the inoculum after 24 h of contact, but still 8.93 × 10^5^ CFU/mL higher than the inoculum at “zero” time. These findings indicate that while the lignin used demonstrated antibacterial activity against *S. aureus*—as evidenced by the bacterial reduction observed in the CO_10%, CO_20%, and CO_30% samples—the same degree of bacterial reduction was not achieved in the PES_10% sample.

Regarding *K. pneumoniae,* no significant antibacterial effect was observed in the PES_10% sample, as the bacterial count increased by more than 10^7^ CFU/mL, when compared to the inoculum at “zero” time. This lack of antibacterial activity against *K. Pneumoniae* is consistent with the conclusions taken on cotton samples and could be attributed to the distinct cell wall structure and inherent resistance mechanisms of this bacteria, when compared to *S. aureus* [12,14].

The antibacterial activity results demonstrated that the lignin-based coating effectively inhibited the growth of Gram-positive *S. aureus* on cotton fabrics, particularly at lignin concentrations of 10% or higher. In contrast, when applied to polyester fabrics, the 10% lignin coating did not achieve a comparable level of bacterial reduction. This observation suggests that the antibacterial efficacy of the lignin-based coating may be influenced by the nature of the textile substrate, potentially due to differences in surface chemistry or topography. Considering that the cotton and polyester fabrics used in this study were structurally distinct (as observed in Figure 1a and Figure 2a), this may affect the interactions between the coating and the underlying material, particularly regarding the accessibility of lignin on the coating surface. For Gram-negative *K. pneumoniae*, no antibacterial activity was observed for both cotton and polyester fabric regardless of the lignin concentration used, which indicates that the lignin tested does not have antibacterial properties against this type of bacteria. These findings align with reports in the literature, which highlight that lignin, being a heterogeneous polymer, can exhibit varying antibacterial activities depending on its origin, extraction processes, parameters applied during isolation, and the type of bacteria. Dong et al. [14] evaluated the antibacterial activity of lignin extracted from residues of corn stover and found that it showed antibacterial effects against Gram-positive bacteria (*Listeria monocytogenes* and *Staphylococcus aureus*), but not against Gram-negative bacteria (*Escherichia coli* O157 and *Salmonella* Enteritidis) or bacteriophage MS2. Additionally, Solihat et al. [20] coated ramie fabrics with lignin from Acacia crassicarpa black liquor, observing that inhibition zones varied depending on the type of Gram-positive bacteria tested. Nevertheless, regarding the antibacterial properties of lignin-coated fabrics, the research is still very limited, with no studies being reported evaluating the antibacterial activity of fabrics coated only with lignin-based PU coatings. Thus, this study showed that the lignin-based PU coatings tested displayed antibacterial efficacy against Gram-positive bacteria, which are more susceptible due to their less complex cell wall structure. Conversely, Gram-negative bacteria are known for their higher resistance to antibacterial agents, which may explain the absence of activity against *K. pneumoniae* [12,14]. Future studies should explore how the differences in substrate properties, like surface chemistry or structure, affect the coating’s performance and consider adjusting the formulation or application process to improve antibacterial efficacy on various fabrics.

### 2.4. Durability of Coated Fabrics to Domestic Washing and Hot Pressing

To further explore the potential applicability of the coated fabrics, an evaluation of their durability was also conducted. Given the obtained results, particularly regarding antibacterial performance, the durability study was focused only on CO_10%, since it was one of the samples that exhibited the highest bacterial reduction. The durability assessment involved subjecting the CO_10% sample to two distinct forms of mechanical and thermal stress: repeated domestic washing cycles (1, 5, 10, and 20 cycles) and hot-pressing at 110 °C and 200 °C, for 15 s under a pressure of 4 kPa. These conditions were selected to simulate the typical mechanical and thermal stresses encountered during the textile’s practical use. The primary goal was then to understand the impact of these stresses on the sample’s key properties, namely, its hydrophobic behavior and antibacterial activity, particularly against *Staphylococcus aureus*.

**Figure 7:**
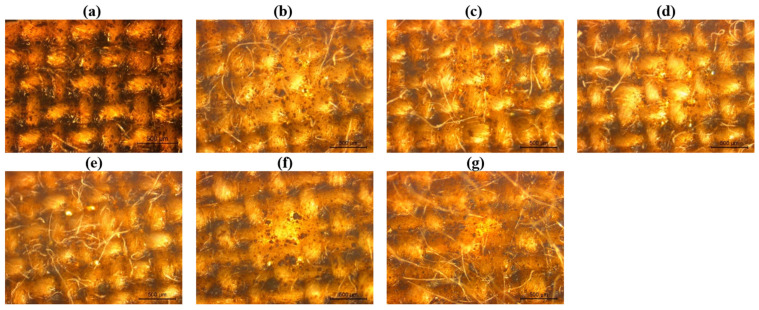
Microscopic images obtained at a total magnification of 50, of the samples: (**a**) CO_10%, (**b**) CO_10%_1, (**c**) CO_10%_5, (**d**) CO_10%_10, (**e**) CO_10%_20, (**f**) CO_10%_110 °C, (**g**) CO_10%_200 °C.

Prior to evaluating the functional properties, a morphological analysis of the surface of the coatings was conducted using optical microscopy to assess whether the applied conditions induced any structural changes. As depicted in Figure 7, some alterations in the surface morphology of the coated cotton fabrics were observed following both washing and hot-pressing treatments, compared to the unaltered CO_10% sample. According to Figure 7b–e, the washing cycles resulted in a pronounced protrusion of fibers from the coating surface, with the number of lignin particles visibly decreasing after the application of 10 washing cycles. This suggests that repeated laundering may have caused mechanical abrasion, leading to partial removal of the coating and consequently to a reduction in the lignin content, which increased fiber exposure. In samples subjected to hot-pressing, fiber protrusion was also observed, particularly in those exposed to 200 °C (Figure 7g). This effect may be attributed to partial softening of the polyurethane polymeric base, which according to the datasheet of Edolan SN, happens around 130–150 °C, allowing fibers to detach more easily from the fabric’s surface due to compression and consequently decompression. However, in contrast to the washing cycles, the amount of lignin on the fabric surface appeared unaffected by hot-pressing, indicating that the thermal treatment did not significantly compromise the integrity of the coating.

**Figure 8:**
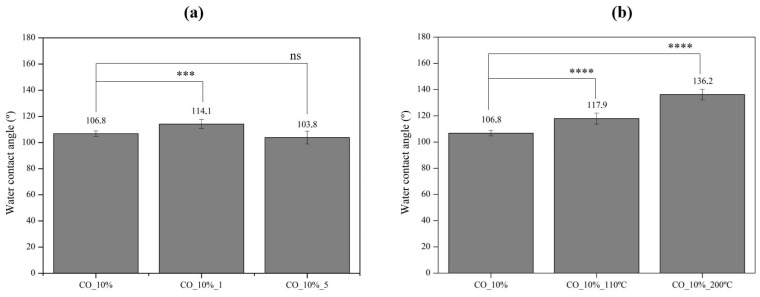
Water contact angle of CO_10% sample subject to (**a**) different domestic washing cycles and (**b**) hot pressing at 110 °C and 200 °C, (*n* = 10, ±SD), ns (non-significant) *p* > 0.05, *** *p* < 0.001, **** *p* < 0.0001 (one-way ANOVA, Šídák’s test).

Nevertheless, to assess with uncertainty the effect of both washing cycles and hot-pressing on the coated cotton fabric properties, the water contact angle was reassessed. As shown in Figure 8a, it was evident that the washing cycles changed the hydrophobicity of the CO_10% samples. Until five washing cycles, the hydrophobicity of the sample was maintained since the contact angles after one and five cycling washes were 114.1° and 103.8°, respectively. However, starting from 10 washing cycles the samples lose completely their hydrophobic behavior, and as soon as the water drop was placed onto the surface of the sample, it was instantly absorbed, which is why these samples are not present in Figure 8a. This could be attributed to the removal or wear of the coating material, which diminishes the overall water-repellent performance of the coated fabric. This tendency aligns with the morphological observations, where fiber protrusion and surface wear were noted after repeated washing, which indicates that the coating is not resistant to washing.

On the other hand, Figure 8b presents the results of the hot-pressing treatment. A significant increase in the contact angle was observed after both 110 °C and 200 °C treatments, with values rising to 117.9° and 136.2°, respectively, which indicated that hot pressing did not decrease the hydrophobicity of the coated samples, but instead increased it, almost reaching values close to the super-hydrophobic behavior (≥150°) [30]. Higher contact angles following the exposure to high temperatures could be explained by the partial softening of the polyurethane polymeric base, which allows for the reorganization of the polymer chains, which potentially enhances the migration of hydrophobic segments to the surface. This reconfiguration likely reduces the surface energy, enhancing the coating surface’s water-repellent properties.

The antibacterial properties of the CO_10% sample against *S. aureus* were also assessed to determine whether exposure to washing or hot-pressing cycles influenced the sample’s antibacterial performance. According to the results presented in Table 5, after undergoing 1, 5, and 10 washing cycles, the bacterial reduction decreased to 92.9%, 86.2%, and 75.8%, respectively. These results indicate that the bacterial count in the treated samples remained lower than that of the inoculum (control). However, when compared to the initial bacterial count at the beginning of the test, the number of bacteria present in the samples after 24 h of contact had increased by approximately 3 × 10^6^ to 10 × 10^6^ CFU/mL, depending on the number of washing cycles applied. For the sample subjected to 20 washing cycles, no antibacterial reduction was observed, suggesting that even in comparison to the control, the bacterial count was higher. This outcome demonstrates that the application of domestic washing cycles significantly compromises the antibacterial properties of the CO_10% sample against *S. aureus*, with a notable decline in efficacy after just one washing cycle. This result is consistent with the aforementioned findings, which demonstrated that the coating lacks wash resistance. Consequently, with the removal of the coating, the concentration of the lignin, the agent responsible for the antibacterial activity, diminished to a level at which the coated fabric no longer exhibited antibacterial properties against *S. aureus* like those observed before washing.

In contrast, this effect was not observed in the samples exposed to hot pressing. As shown in Table 5, both hot pressing at 110 °C and 200 °C did not negatively affect the antibacterial performance of the CO_10% sample, which retained reduction values of 96.6% and 97.3%, respectively. These findings are in line with the conclusions drawn previously, where it was found that hot pressing did not alter the amount or structural integrity of the lignin-based coating, thereby preserving the antibacterial effect observed on the CO_10% sample.

Based on durability assessments, it was observed that lignin-coated cotton fabrics exhibit limited wash resistance. Conversely, these coatings show no significant changes under thermal stress. The current literature lacks studies specifically examining the washing durability of lignin-based PU coatings applied to textile fabrics. However, microscopic analyses and the loss of hydrophobic and antibacterial properties previously observed suggested a low affinity between the applied coating and the cotton surface, leading to a rapid coating detachment under washing conditions. Consequently, the removal of the coating results in the loss of lignin in sufficient amounts to reduce the antibacterial properties, after a single washing cycle. Following 10 washing cycles, the inherent hydrophilic behavior of the cotton fabric also re-emerges due to the coating’s detachment. According to the literature, a potential strategy to enhance wash resistance would be incorporating a crosslinker, such as an aliphatic polyisocyanate, to form additional chemical bonds between polymer chains. This modification would yield a denser, more stable, and durable coating, increasing its washing resistance [35].

In summary, this study enabled the development of textiles with combined antibacterial and hydrophobic properties, through the application of lignin-based coatings, especially on cotton fabrics. However, given the durability results, coated cotton fabrics are not suitable for applications demanding wash resistance. Further optimization of the coating formulation, such as the inclusion of a crosslinker, is required for enhanced durability. Alternatively, these lignin-coated fabrics could be utilized in single-use, short-term applications, or those not requiring washing.

## 3. Materials and Methods

### 3.1. Materials

The kraft lignin used in this study was provided by Suzano (São Paulo, Brazil). The polyurethane polymeric base employed in the preparation of coating formulations, Edolan SN and Thickener A02, were purchased from ADI Center Portugal, S.A (Santo Tirso, Portugal). Coatings were applied in desized and scoured cotton fabric (plain weave, 121 g/m^2^, warp and weft density = 32/cm and 32/cm), acquired from Lameirinho–Indústria Têxtil, S.A. (Guimarães, Portugal) and polyester fabric (plain weave, 272 g/m^2^, warp and weft density = 13/cm and 13/cm), acquired from Playvest (Braga, Portugal).

### 3.2. Methods

#### 3.2.1. Preparation of Lignin-Based Coating Formulations

The coating formulations were prepared by incorporating lignin powder, previously dried at 50 °C for 24 h, at varying concentrations of 0%, 5%, 10%, 20%, and 30% (*w*/*v*), to Edolan SN, a polyurethane dispersion. To ensure homogeneous dispersion, the specified amounts of lignin were slowly added to Edolan SN and mixed under mechanical stirring for 30 min, approximately. Before applying to cotton and polyester fabrics, Thickener A02 was added to the formulation. Due to the thickening effect of the lignin when dispersed in Edolan SN, the amount of thickener used in the formulations was adjusted based on lignin concentration, ranging from 1% to 0.1% (*w*/*v*). Higher lignin concentrations required less additional thickener to maintain the desired viscosity of the coating formulation.

#### 3.2.2. Application of Lignin-Based Coating Formulations to Cotton and Polyester Fabrics

**Figure 9:**
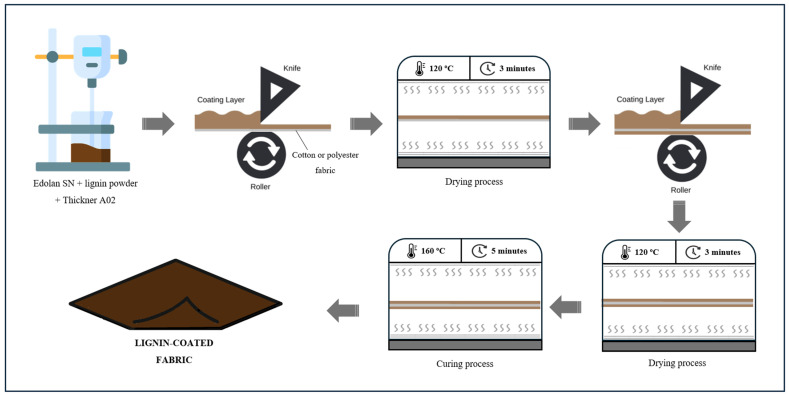
Schematic representation of preparation and application of lignin-based coating formulations to cotton or polyester fabric.

Cotton and polyester fabric samples (30 cm × 40 cm) were coated using a laboratory-scale knife-coating machine (Mathis AG, Rutisbergstrasse, Switzerland). The fabric was first fixed and tensioned on a metallic frame integrated into the equipment. The prepared coating formulations were then applied to the fabric, and the excess material was removed using a knife set at a distance from the surface of the fabric of approximately 50 μm, using a leveling system. The samples were then oven-dried at 120 °C for 3 min. The coating procedure was subsequently repeated on the opposite side of the fabric. After coating both sides, the samples underwent thermal curing at 160 °C for 5 min to ensure adhesion of the coating. According to the lignin content in the coatings, the cotton samples were coded as CO_0%, CO_5%, CO_10%, CO_20%, and CO_30%, and the polyester samples were coded as PES_0%, PES_5%, PES_10%, PES_20%, and PES_30%. A schematic representation of the preparation and application of lignin-based coating formulations to cotton and polyester fabrics is presented in Figure 9.

#### 3.2.3. Microscopic, Chemical, and Thermal Characterization of Coated Fabrics

To evaluate the distribution of the lignin on the surface of the coated fabrics, the samples were analyzed using a Leica DM750 M optical microscope equipped with a high-definition camera. For each sample, at least five distinct areas were examined at a total magnification of 50, providing a representative assessment of surface morphology and lignin distribution. The Fourier Transform Infrared Spectroscopy (FTIR) coupled with the Attenuated Reflection (ATR) was employed to analyze the chemical composition of the coated fabrics using an IRAffinity-1S, SHIMADZU spectrophotometer (Kyoto, Japan) with a diamond crystal. Spectra were collected at a resolution of 8 cm⁻^1^ over 45 scans, covering a spectral range from 4000 to 400 cm⁻^1^. To assess the thermal stability of the coated samples, a thermogravimetric analysis (TGA) was performed using an STA 700 analyzer (HITACHI, Tokyo, Japan). The measurements were conducted under a nitrogen atmosphere over a temperature range of 25 °C to 600 °C, with a heating rate of 10 °C/min.

#### 3.2.4. Air and Water Vapor Permeability Assessment

To evaluate the ability of functionalized fabrics to facilitate air exchange and moisture transmission, air permeability and water vapor permeability tests were performed, following adaptations of the ISO 9237:1995 [43] and BS 7209:1990 [44] standards, respectively. Air permeability was measured using a TEXTEST Instruments Air Permeability Tester III FX 3300 (Sonnenbergstrasse, Switzerland), applying a pressure of 200 Pa at ten equidistant points over a test area of 20 cm^2^.

For water vapor permeability (WVP), the coated fabric samples were placed on top of cylindrical cups containing 46 mL of deionized water at ambient conditions (20–24 °C, 65% relative humidity). The evaporation of water through the samples was determined by weighing the cup before and after the testing period and for each sample, measurements were conducted in triplicate. Using standard fabric for water vapor tester from SDL ATLAS as a reference, the water vapor permeability (*WVP*) and the *WVP* index (*I*) were then determined, according to Equations (1) and (2), respectively:

(1) $$\mathrm{WVP}=\frac{24\Delta W}{A\Delta t}$$

(2) $$I=\frac{{\mathrm{WVP}}_{s}}{{\mathrm{WVP}}_{r}}$$

where ∆W is the difference in water mass (g) before and after the test, A is the internal area of the cup (mm^2^), ∆t is the exposure time (h), WVP_s_ is the water vapor permeability of the sample, and WVP_r_ is the water vapor permeability of the reference fabric. Uncoated cotton and polyester fabric, coded as CO and PES, respectively, were also analyzed to evaluate the impact of the coating on the fabric’s properties.

#### 3.2.5. Water Contact Angle Evaluation

The water contact angle (WCA) of the samples was measured using a Contact Angle System OCA 15 goniometer (DataPhysics Instruments, Filderstadt, Germany), equipped with a high-resolution digital camera. A 5 μL droplet of distilled water was dispensed at a flow rate of 10 μL/s onto the surface of the fabric samples. Measurements were recorded at ten different locations on each sample, and the mean values along with standard deviations were calculated. The wettability of the samples was classified based on the WCA values: samples with a WCA of less than 90° were considered hydrophilic; those with angles between 90° and 150° were classified as hydrophobic; and angles above 150° indicated superhydrophobic behavior [40].

#### 3.2.6. Investigation of Antibacterial Performance of Coated Fabrics

The antibacterial activity of both uncoated and coated fabrics was evaluated according to the adaptation of ASTM E2149-2013 [45]. The analysis was conducted against the Gram-negative bacteria *Klebsiella pneumoniae* (ATCC 4352) and the Gram-positive bacteria *Staphylococcus aureus* (ATCC 6538).

For the evaluation, 1 g of each fabric sample was incubated with a bacterial suspension with a concentration of 1.5–3.0 × 10^5^ CFU/mL, under agitation in a temperature-controlled shaker set to 37 °C. A control bacterial suspension without any fabric sample served as a reference. Following a 24 h incubation period, 1 mL of suspension was collected, and the bacterial count was determined. The tests were conducted in duplicate to ensure reliability. The average of the two duplicates was converted into colony-forming units per milliliter (CFU/mL), when the bacterial counts of the duplicates coincided by 15%.

The reduction in bacterial count (*R*) was calculated using the Equation (3):

(3) $$R\left(\%\right)=\frac{B-A}{B}\times 100$$

where *A* represents the number of bacteria (CFU/mL) in the fabric samples after 24 h of incubation and *B* denotes the number of bacteria (CFU/mL) in the reference inoculum (without any fabric samples) after 24 h of incubation.

#### 3.2.7. Durability Assessment of Coated Fabrics

To evaluate the durability of the coatings, the coated fabrics were subjected to multiple domestic washing cycles and hot pressing at different temperatures. At this point, only the durability of coatings applied to cotton fabric was assessed.

Domestic washing durability test: The washing durability of the coated fabrics was evaluated according to an adaptation of ISO 105-C06:2010 [46]. The fabrics were placed in a domestic washing machine and subjected to washing cycles lasting 1 h at 40 °C, using approximately 4 g/L of ECE detergent, along with knitting ballasts to enhance mechanical friction during the washing process. To assess the coating’s durability, samples were subjected to 1, 5, 10, and 20 washing cycles. According to the number of cycles applied, the samples were coded as CO_*x*%_1, CO_*x*%_5, CO_*x*%_10, and CO_*x*%_20, with *x* being the amount of lignin applied to the coating.

Hot-pressing durability Test: The resistance of the coatings to hot pressing was evaluated following an adaptation of ISO 105-X11:1994 [47]. The test was conducted using a press where the samples were placed under specific conditions. In accordance with the standard, only the upper plate of the press was heated, while the lower plate remained deactivated. Additionally, a thermal insulator (wool felt) and a cotton cloth were positioned beneath the test samples. Two pressing conditions were applied to evaluate the effect of hot pressing on the samples: temperatures of 110 °C and 200 °C applied for 15 s under a pressure of 4 kPa. After each condition, the samples were removed from the press and left to cool to room temperature. According to the temperature applied during the hot-pressing, the samples were coded as CO_*x*%_110 °C and CO_*x*%_200 °C, with *x* being the amount of lignin applied on the coating.

Following these tests, the surface morphology, water contact angle, and antibacterial properties were reassessed according to the methodology previously described.

#### 3.2.8. Statistical Analysis

The statistical analysis of the results obtained throughout this paper was performed using the software GraphPad Prism 6.01. The results are presented as the average of the replicates performed, and the respective standard deviation. The results were analyzed using a one-way ANOVA test and multiple comparisons were performed using Šídák’s test. A critical value for significance of *p* < 0.05 was used throughout the study.

## 4. Conclusions

This study evaluated the potential of unmodified lignin, sourced from the paper industry, through its incorporation into polyurethane-based coatings applied to both sides of cotton and polyester fabrics, via knife coating. Coatings with varying lignin concentrations (0%, 5%, 10%, 20%, and 30% *w*/*v*) were homogeneously applied on both cotton and polyester fabrics, with lignin particles distributed across the entire fabric’s surface, which was confirmed through optical microscopy. Independent of lignin concentration, the application of all coatings effectively blocked the air gaps within the fabrics, leading to a significant reduction in both air permeability and the WVP index. For coated cotton samples, air permeability and WVP index values decreased to below 3 L/m^2^/s and 45%, respectively, while for coated polyester samples, these values were reduced to below 8 L/m^2^/s and 12%, respectively. On the other hand, the low surface energy of the PU polymeric base combined with the hydrophobic properties associated with lignin, allowed the development of hydrophobic cotton and polyester fabrics, with WCA achieving 112.6° and 110.3°, respectively, when 30% of lignin was applied. Additionally, the antibacterial evaluations also revealed that lignin-based coatings successfully conferred antibacterial properties, particularly against Gram-positive *S. aureus*. Notably, concentrations of lignin equal to or superior to 10% in the coating resulted in bacterial reductions surpassing 96%, highlighting the potential of lignin as an antibacterial agent. However, the antibacterial efficacy on polyester fabrics was reduced, as demonstrated by a bacterial reduction of 84.3%, with 10% lignin coatings. This disparity suggests that the antibacterial performance of lignin-based coatings may depend on the substrate’s surface chemistry or structure since the cotton and polyester fabrics used were structurally different. Additionally, no antibacterial activity was observed against *K. pneumoniae* (Gram-negative) for either fabric, likely due to the inherent resistance of this microorganism.

While the lignin-based coatings successfully conferred antibacterial and hydrophobic properties to cotton fabrics, these functionalities were significantly diminished after washing, with antibacterial and hydrophobic properties lost after 1 and 10 washing cycles, respectively. This outcome indicates that the applied coating begins to be removed from the cotton surface when washed, resulting in a loss of the acquired functionalities. Nevertheless, the coatings exhibited remarkable stability under thermal stress at 110 °C and 200 °C, preserving their functional attributes.

Therefore, this study suggests that lignin-based coatings hold potential for the development of antibacterial and water-repellent textiles suitable for single-use or short-term applications, particularly where washing resistance is not a critical requirement. Further research optimizing coating’s formulation, such as crosslinker addition, could enhance the washing durability for broader applications.

## Figures and Tables

**Table 1:** Air permeability (*n* = 10, ±SD) and water vapor permeability index (*I*) (*n* = 3, ±SD) of uncoated and coated cotton fabrics, **** *p* < 0.0001 (one-way ANOVA, Šídák’s test).

	CO	CO_0%	CO_5%	CO_10%	CO_20%	CO_30%
**Air permeability (L/m^2^/s (200 Pa))**	679.8 ± 18.5	1.8 ± 0.1 ****	1.3 ± 0.1 ****	1.3 ± 0.1 ****	2.7 ± 0.1 ****	2.2 ± 0.1 ****
***I*** **(%)**	100.0 ± 0.2	41.9 ± 0.2 ****	37.5 ± 1.9 ****	33.7 ± 1.2 ****	44.2 ± 1.8 ****	36.7 ± 1.3 ****

**Table 2:** Air permeability (*n* = 10, ±SD) and water vapor permeability index (*I*) (*n* = 3, ±SD) of uncoated and coated polyester fabrics, **** *p* < 0.0001 (one-way ANOVA, Šídák’s test).

	PES	PES_0%	PES_5%	PES_10%	PES_20%	PES_30%
**Air permeability (L/m^2^/s (200 Pa))**	57.0 ± 3.1	5.8 ± 0.9 ****	2.3 ± 0.4 ****	3.1 ± 0.4 ****	2.6 ± 0.3 ****	7.8 ± 0.9 ****
***I*** **(%)**	84.1 ± 1.5	9.9 ± 0.0 ****	11.6 ± 0.7 ****	11.3 ± 1.2 ****	9.2 ± 0.7 ****	12.0 ± 0.2 ****

**Table 3:** Antibacterial activity of uncoated and coated cotton fabrics against *Staphylococcus aureus* (ATCC 6538) and *Klebsiella pneumoniae* (ATCC 4352), (all samples were tested in duplicate).

	Number of Bacteria *Staphylococcus aureus* (ATCC 6538) (CFU/mL)			*R* (%)
	Time “0” in the Inoculum	After 24 h of Contact with the Inoculum (Control)	After 24 h of Contact with the Sample	
**CO**	1.93 × 10^5^	6.73 × 10^6^	5.95 × 10^6^	11.8
**CO_0%**	1.73 × 10^5^	6.73 × 10^6^	2.24 × 10^7^	0
**CO_5%**	1.99 × 10^5^	7.27 × 10^6^	1.12 × 10^6^	84.6
**CO_10%**	1.99 × 10^5^	7.27 × 10^6^	1.95 × 10^5^	97.3
**CO_20%**	2.21 × 10^5^	6.10 × 10^6^	2.03 × 10^5^	96.7
**CO_30%**	2.21 × 10^5^	6.10 × 10^6^	1.90 × 10^5^	96.9
	**Number of bacteria *Klebsiella pneumoniae* (ATCC 4352) (CFU/mL)**			
**CO**	2.43 × 10^5^	1.65 × 10^7^	8.93 × 10^6^	45.7
**CO_0%**	2.34 × 10^5^	7.77 × 10^6^	1.24 × 10^7^	0
**CO_5%**	1.95 × 10^5^	4.40 × 10^7^	3.67 × 10^7^	17.0
**CO_10%**	2.34 × 10^5^	7.77 × 10^6^	1.61 × 10^7^	0
**CO_20%**	1.95 × 10^5^	4.40 × 10^7^	2.17 × 10^7^	51.0
**CO_30%**	1.95 × 10^5^	4.40 × 10^7^	2.15 × 10^7^	51.0

**Table 4:** Antibacterial activity of uncoated and coated polyester fabrics against *Staphylococcus aureus* (ATCC 6538) and *Klebsiella pneumoniae* (ATCC 4352) (all samples were tested in duplicate).

	Number of bacteria *Staphylococcus aureus* (ATCC 6538) (CFU/mL)			*R* (%)
	Time “0” in the Inoculum	After 24 h of Contact with the Inoculum (Control)	After 24 h of Contact with the Sample	
**PES**	1.57 × 10^5^	2.9 × 10^7^	2.23 × 10^7^	23.1
**PES_0%**	1.97 × 10^5^	6.93 × 10^6^	1.29 × 10^7^	0
**PES_10%**	1.97 × 10^5^	6.93 × 10^6^	1.09 × 10^6^	84.3
	**Number of bacteria *Klebsiella pneumoniae* (ATCC 4352) (CFU/mL)**			
**PES**	2.43 × 10^5^	1.65 × 10^7^	1.65 × 10^7^	0
**PES_0%**	2.22 × 10^5^	3.47 × 10^7^	2.77 × 10^7^	20.0
**PES_10%**	2.22 × 10^5^	3.47 × 10^7^	2.23 × 10^7^	35.8

**Table 5:** Antibacterial activity against *Staphylococcus aureus* (ATCC 6538) of CO_10% sample before and after the application of washing cycles (1, 5, 10, and 20) and hot pressing at 110 °C and 200 °C (all samples were tested in duplicate).

	Number of Bacteria *Staphylococcus aureus* (ATCC 6538) (CFU/mL)			*R* (%)
	Time “0” in the Inoculum	After 24 h of Contact with the Inoculum (Control)	After 24 h of Contact with the Sample	
**CO_10%**	1.99 × 10^5^	7.27 × 10^6^	1.95 × 10^5^	97.3
**CO_10%_1**	1.69 × 10^5^	4.1 × 10^7^	2.89 × 10^6^	92.9
**CO_10%_5**	1.69 × 10^5^	4.1 × 10^7^	5.67 × 10^6^	86.2
**CO_10%_10**	1.69 × 10^5^	4.1 × 10^7^	9.93 × 10^6^	75.8
**CO_10%_20**	1.87 × 10^5^	6.93 × 10^6^	7.43 × 10^6^	0
**CO_10%_110 °C**	1.87 × 10^5^	6.93 × 10^6^	2.35 × 10^5^	96.6
**CO_10%_200 °C**	1.87 × 10^5^	6.93 × 10^6^	1.88 × 10^5^	97.3

## Data Availability

Data are contained within the article.

## References

[1] Afonso T.B., Bonifácio-Lopes T., Costa E.M., Pintado M.E. (2023). Phenolic Compounds from By-Products for Functional Textiles. Materials.

[2] Gulati R., Sharma S., Sharma R.K. (2022). Antimicrobial textile: Recent developments and functional perspective. Polym. Bull..

[3] Morais D.S., Guedes R.M., Lopes M.A. (2016). Antimicrobial approaches for textiles: From research to market. Materials.

[4] Abdouramane N., Sibiescu D., Efeze N.D., Noah P.M.A., Betene F.E., Gomdjé Valery H. (2023). Analysis of bio-based antimicrobial textiles: A review. Environ. Eng. Manag. J..

[5] Bibi A., Afza G., Afzal Z., Farid M., Sumrra S.H., Hanif M.A., Jinadasa B.K.K.K., Zubair M. (2024). Synthetic vs. natural antimicrobial agents for safer textiles: A comparative review. RSC Adv..

[6] Raman A., Sankar A., Abhirami S.D., Anilkumar A., Saritha A. (2022). Insights into the Sustainable Development of Lignin-Based Textiles for Functional Applications. Macromol. Mater. Eng..

[7] Das A.K., Mitra K., Conte A.J., Sarker A., Chowdhury A., Ragauskas A.J. (2024). Lignin–A green material for antibacterial application—A review. Int. J. Biol. Macromol..

[8] Wang Y.Y., Meng X., Pu Y., Ragauskas A.J. (2020). Recent advances in the application of functionalized lignin in value-added polymeric materials. Polymers.

[9] Reyes D.C., Ma Z., Romero J.J. (2024). The Antimicrobial Properties of Technical Lignins and Their Derivatives—A Review. Polymers.

[10] Rinaldi R., Jastrzebski R., Clough M.T., Ralph J., Kennema M., Bruijnincx P.C., Weckhuysen B.M. (2016). Paving the Way for Lignin Valorisation: RecentAdvances in Bioengineering, Biorefining and Catalysis. Angew. Chem..

[11] Zheng L., Lu G., Pei W., Yan W., Li Y., Zhang L., Huang C., Jiang Q. (2021). Understanding the relationship between the structural properties of lignin and their biological activities. Int. J. Biol. Macromol..

[12] Li K., Zhong W., Li P., Ren J., Jiang K., Wu W. (2023). Antibacterial mechanism of lignin and lignin-based antimicrobial materials in different fields. Int. J. Biol. Macromol..

[13] Ndaba B., Roopnarain A., Daramola M.O., Adeleke R. (2020). Influence of extraction methods on antimicrobial activities of lignin-based materials: A review. Sustain. Chem. Pharm..

[14] Dong X., Dong M., Lu Y., Turley A., Jin T., Wu C. (2011). Antimicrobial and antioxidant activities of lignin from residue of corn stover to ethanol production. Ind. Crops Prod..

[15] Huang D., Li R., Xu P., Li T., Deng R., Chen S., Zhang Q. (2020). The cornerstone of realizing lignin value-addition: Exploiting the native structure and properties of lignin by extraction methods. Chem. Eng. J..

[16] Yun J., Wei L., Li W., Gong D., Qin H., Feng X., Li G., Ling Z., Wang P., Yin B. (2021). Isolating High Antimicrobial Ability Lignin From Bamboo Kraft Lignin by Organosolv Fractionation. Front. Bioeng. Biotechnol..

[17] Gordobil O., Herrera R., Yahyaoui M., İlk S., Kaya M., Labidi J. (2018). Potential use of kraft and organosolv lignins as a natural additive for healthcare products. RSC Adv..

[18] Jin Y., Lin J., Cheng Y., Lu C. (2021). Lignin-based high-performance fibers by textile spinning techniques. Materials.

[19] Baysal G. (2023). Mechanical and UV protection performances of polylactic acid spunlace nonwoven fabrics coated by eco-friendly lignin/water-borne polyurethane composite coatings. J. Text. Inst..

[20] Solihat N.N., Purwanti T., Husna N., Oktaviani M., Zulfiana D., Fatriasari W., Nawawi D.S. (2024). Capability lignin from Acacia crassicarpa black liquor as an environmentally benign antibacterial agent to produce antibacterial and hydrophobic textiles. Bioresour. Technol..

[21] Sunthornvarabhas J., Liengprayoon S., Suwonsichon T. (2017). Antimicrobial kinetic activities of lignin from sugarcane bagasse for textile product. Ind. Crops Prod..

[22] Sunthornvarabhas J., Liengprayoon S., Lerksamran T., Buratcharin C., Suwonsichon T., Vanichsriratana W., Sriroth K. (2019). Utilization of Lignin Extracts from Sugarcane Bagasse as Bio-based Antimicrobial Fabrics. Sugar Tech.

[23] Bhushan S., Kumar A., Singh N., Sheikh J. (2020). Functionalization of wool fabric using lignin biomolecules extracted from groundnut shells. Int. J. Biol. Macromol..

[24] Liu J., Cui X., Qu Y., Su X., Li X., Qi P., Li H., Gu X., Sun J., Zhang S. (2024). Durable Flame-Retardant, Anti-UV, Photothermal, and Antibacterial Cotton Fabrics Based on Lignin and Ammonium Polyphosphate. ACS Sustain. Chem. Eng..

[25] Juikar S.J., Nadanathangam V. (2020). Microbial Production of Nanolignin from Cotton Stalks and Its Application onto Cotton and Linen Fabrics for Multifunctional Properties. Waste Biomass Valorization.

[26] Safi K., Kant K., Bramhecha I., Mathur P., Sheikh J. (2020). Multifunctional modification of cotton using layer-by-layer finishing with chitosan, sodium lignin sulphonate and boric acid. Int. J. Biol. Macromol..

[27] Marković D., Petkovska J., Mladenovic N., Radoičić M., Rodriguez-Melendez D., Ilic-Tomic T., Radetić M., Grunlan J.C., Jordanov I. (2023). Antimicrobial and UV protective chitosan/lignin multilayer nanocoating with immobilized silver nanoparticles. J. Appl. Polym. Sci..

[28] Petkovska J., Mladenovic N., Marković D., Radoičić M., Vest N.A., Palen B., Radetić M., Grunlan J.C., Jordanov I. (2022). Flame-Retardant, Antimicrobial, and UV-Protective Lignin-Based Multilayer Nanocoating. ACS Appl. Polym. Mater..

[29] Baysal G. (2024). Sustainable polylactic acid spunlace nonwoven fabrics with lignin/zinc oxide/water-based polyurethane composite coatings. Int. J. Biol. Macromol..

[30] Sha X., Chen L., Jia Y., Zhao H., Zuo S., Yuan P., Chen G. (2024). Preparation and properties of sustainable superhydrophobic cotton fabrics modified with lignin nanoparticles, tannic acid and methyltrimethoxysilane. Chem. Eng. J..

[31] Li M., Prabhakar M.N., Song J.I. (2024). Effect of synthesized lignin-based flame retardant liquid on the flame retardancy and mechanical properties of cotton textiles. Ind. Crops Prod..

[32] Zhang Y., Li T.T., Lou C.W., Lin J.H. (2022). Facile method for tent fabrics with eco-friendly/durable properties using waterborne polyurethane/lignin: Preparation and evaluation. J. Ind. Text..

[33] Fodil Cherif M., Trache D., Brosse N., Benaliouche F., Tarchoun A.F. (2020). Comparison of the Physicochemical Properties and Thermal Stability of Organosolv and Kraft Lignins from Hardwood and Softwood Biomass for Their Potential Valorization. Waste Biomass Valorization.

[34] Pasichnyk M., Gaálová J., Minarik P., Václavíková M., Melnyk I. (2022). Development of polyester filters with polymer nanocomposite active layer for effective dye filtration. Sci. Rep..

[35] De Smet D., Wéry M., Uyttendaele W., Vanneste M. (2021). Bio-based waterborne PU for durable textile coatings. Polymers.

[36] Küçük M., Öveçoğlu M.L. (2018). Surface modification and characterization of polyester fabric by coating with low temperature synthesized ZnO nanorods. J. Solgel Sci. Technol..

[37] Rahman Bhuiyan M.A., Wang L., Shaid A., Shanks R.A., Ding J. (2019). Polyurethane-aerogel incorporated coating on cotton fabric for chemical protection. Prog. Org. Coat..

[38] Baysal G. (2024). Investigation of Barrier Effectiveness and Comfort Properties of Biodegradable PLA Nonwoven Fabrics Coated with Unmodified Lignin/Water-Borne Polyurethane Composite Coatings. Hittite J. Sci. Eng..

[39] Ghezal I., Moussa A., Ben Marzoug I., El-Achari A., Campagne C., Sakli F. (2022). Investigating Waterproofness and Breathability of a Coated Double-Sided Knitted Fabric. Coatings.

[40] Melki S., Biguenet F., Dupuis D. (2019). Hydrophobic properties of textile materials: Robustness of hydrophobicity. J. Text. Inst..

[41] Crouvisier-Urion K., Bodart P.R., Winckler P., Raya J., Gougeon R.D., Cayot P., Domenek S., Debeaufort F., Karbowiak T. (2016). Biobased Composite Films from Chitosan and Lignin: Antioxidant Activity Related to Structure and Moisture. ACS Sustain. Chem. Eng..

[42] Liu X., Gao C., Fu C., Xi Y., Fatehi P., Zhao J.R., Wang S., Gibril M.E., Kong F. (2022). Preparation and Performance of Lignin-Based Multifunctional Superhydrophobic Coating. Molecules.

[43] (1995). Textiles—Determination of the Air Permeability of Fabrics to Air.

[44] (1990). Specification for Water Vapour Permeable Apparel Fabrics.

[45] (2013). Standard Test Method for Determining the Antimicrobial Activity of Immobilized Antimicrobial Agents Under Dynamic Contact Conditions.

[46] (2010). Textiles—Tests for Colour Fastness—Part C06: Colour Fastness to Domestic and Commercial Laundering.

[47] (1994). Textiles—Tests for Colour Fastness—Part X11: Colour Fastness to hot Pressing.

